# A gravimetric assessment of the Gotthard Base Tunnel geological model: insights from a novel gravity terrain-adaptation correction and rock physics data

**DOI:** 10.1186/s00015-022-00422-z

**Published:** 2022-11-11

**Authors:** M. Scarponi, G. Hetényi, L. Baron, U. Marti

**Affiliations:** 1 Institute of Earth Sciences, University of Lausanne, Lausanne, Switzerland; 2 Institute of Geophysics, Czech Academy of Sciences, Prague, Czech Republic; 3 Federal Office of Topography (Swisstopo), Wabern, Switzerland

**Keywords:** Gotthard Base Tunnel, Gravity modelling, Geological model, Central Alps, Relative gravity measurements, Density-dependent terrain-adaptation correction

## Abstract

The Gotthard Base Tunnel (GBT) is a 57 km long railway tunnel, constructed in the Central Alps in Switzerland and extending mainly North–South across numerous geological units. We acquired 80 new gravity data points at the surface along the GBT profile and used 77 gravity measurements in the tunnel to test and constrain the shallow crustal, km-scale geological model established during the tunnel construction. To this end, we developed a novel processing scheme, which computes a fully 3D, density-dependent gravity terrain-adaptation correction (TAC), to consistently compare the gravity observations with the 2D geological model structure; the latter converted into a density model. This approach allowed to explore and quantify candidate rock density distributions along the GBT modelled profile in a computationally-efficient manner, and to test whether a reasonable fit can be found without structural modification of the geological model. The tested density data for the various lithologies were compiled from the SAPHYR rock physical property database. The tested models were evaluated both in terms of misfit between observed and synthetic gravity data, and also in terms of correlation between misfit trend and topography of the target profile. The results indicate that the locally sampled densities provide a better fit to the data for the considered lithologies, rather than density data averaged over a wider set of Alpine rock samples for the same lithology. Furthermore, using one homogeneous and constant density value for all the topographic corrections does not provide an optimal fit to the data, which instead confirms density variations along the profile. Structurally, a satisfactory fit could be found without modifying the 2D geological model, which thus can be considered gravimetry-proof. From a more general perspective, the gravity data processing routines and the density-dependent corrections developed in this case study represent a remarkable potential for further high-resolution gravity investigations of geological structures.

**Supplementary Information:**

The online version contains supplementary material available at 10.1186/s00015-022-00422-z.

## Introduction

The Gotthard Base Tunnel (GBT) is a 57 km long railway tunnel, currently the longest on Earth, constructed in the Central Alps in Switzerland and extending mainly North–South (at ~ 8.77$^\circ$ E and from ~ 47.37 N$^\circ$ to ~ 46.84 N$^\circ$). Locally, it gets as much as 2500 m below the surface in the areas of higher topography, which ranges between 300 and 3000 m above sea level in the area. The tunnel itself runs at ~ 300–500 m above sea level and crosses numerous important tectonic units, mainly belonging to the European continental crust (e.g. Schmid et al., 2017) and deformed after the Alpine collision (35–30 Ma, Schmid et al., 1996): the Aar Massif, the Tavetsch Massif, the Urseren-Garvera Zone, the Gotthard Massif and the Penninic Gneiss Zone.

The surface and the tunnel surveys related to the construction provided an unprecedented, sub-km-scale and “inside” view of the geological structure of the very shallow crust along the GBT profile (Guntli et al., 2016, Fig. 1). A rich amount of geotechnical data was collected during the tunnel construction and the subsequent operation phases (e.g. Loew et al., 2015). The tunnel crosses several geological units, in structurally vertical to sub-vertical position, with their strikes mainly along the WSW-ENE direction.

**Fig. 1:**
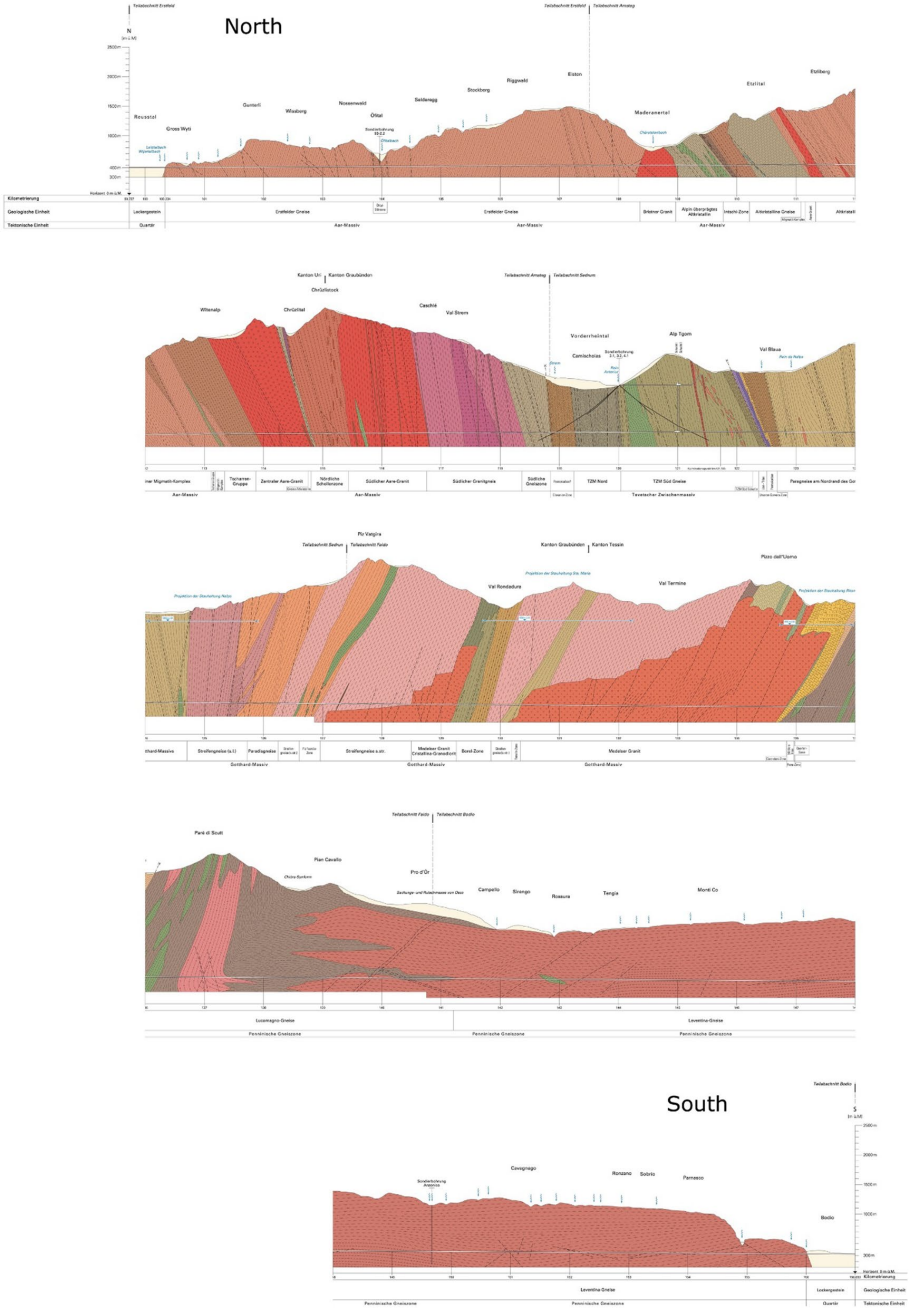
The GBT geological profile, from Guntli et al. (2016). For the sake of graphical representation, the profile has been split into five sections from North to South. The associated legend, describing all the units, is reported in Guntli et al. (2016) in paper format only

During the tunnel construction, relative gravity data was measured by swisstopo (Swiss Federal Office of Topography) inside the tunnel with an average spacing of ~ 800 m, with the use of a Scintrex CG-5 relative gravimeter (i.e. measuring local gravity acceleration with high accuracy). In addition to this data, the Swiss Atlas of Physical Properties of Rocks project (SAPHYR, Zappone & Kissling, 2021) provides a series of rock density data, obtained from laboratory analysis of rock samples collected in the same area.

By combining these datasets, the GBT area constitutes an ideal natural laboratory to test the resolving capabilities of gravity data analysis and the geological units’ density and/or geometry along the GBT profile. Within this framework, the compiled observations span a wide range of spatial scales: the GBT geological model presents sub-km- and km-scale lithological units, interpolated between surface and tunnel field observations; the SAPHYR catalog is based on the analysis of 1-to-10-cm-scale rock samples; eventually, already-existing and newly-performed gravimetric surveys provide larger-scale, indirect constraints on the subsurface density distribution along the GBT profile.

The main goal of our work is to compare and test against each other, from a gravimetric perspective, these complementary geophysical and geological datasets, as their mutual compatibility was never tested before in the area of the GBT, nor should be taken for granted. Is there a lithologically-plausible density distribution, which can explain gravity data without modifying the GBT profile geometries, or structural corrections are warranted in this latter? To verify these, we developed a novel routine to compare the observed gravity with synthetics, based on SAPHYR density data and the GBT geological model.

In the literature of gravimetry, several methods have been suggested to estimate the optimal reduction density for a surveyed topographic profile, via gravity measurement analysis (e.g. Fukao et al., 1981; Nettleton, 1939, 1976; Parasnis, 1952). Nettleton’s method (Nettleton, 1939, 1976) is perhaps one of the most known approaches. It is based on the assumption that the Bouguer gravity anomaly profile, computed for a set of densely spaced gravity measurements along a given topographic profile, should not be correlated at all with the topography when the correct reduction density is applied. Hence, this method allows to set up a trial-and-error search for the best reduction density value, obtained when the correlation between topography and Bouguer gravity anomaly of surface data points approaches zero. The so-obtained density value can be then regarded as the best estimate of the average density along the surveyed topographic profile.

More recently, Zahorec et al. (2019) performed Bouguer gravity anomaly residual modelling, combining surface gravity data with underground-tunnel gravity data; the latter used as an additional constraint on the topographic reduction density choice. In general, underground gravity measurements, either from tunnels (e.g. Capuano et al., 1998; Zahorec & Papčo, 2018) and/or from mines and boreholes (e.g. Hammer, 1950; Hinze et al., 1978; Hussain et al., 1981) have proven to be a valuable and non-invasive source of complementary information when targeting shallow crustal density contrasts below the surface.

The overwhelming majority of these approaches initially makes use of a constant, homogeneous reduction density, which, on its own, is not likely to represent reality in a complex area such as the Alps. At a second stage, the effects of known density heterogeneities are taken into account, including their extent below and/or above sea level, and the Bouguer gravity anomaly is usually modeled to constrain the subsurface properties in terms of differential density distribution. While this procedure would certainly hold valid in our case, we developed an alternative and novel approach for the sake of efficiency and convenience, in terms of both practical implementation and computational costs.

To this end, and as explained below in detail, the lithological units of the GBT geological model profile have been digitized, and used as reference geometries to define 2D density models, the latter based on the SAPHYR catalog density data. The so-obtained 2D density models have been used in the subsequent gravity forward modelling, and compared with the observed gravity data.

While various tools and codes have been developed to perform 3D gravity modeling by other authors (e.g. Schmidt et al. 2011), we decided to work and model gravity observations in 2D, as the high-resolution, km-to-sub-km-scale GBT geological model is available only along the GBT profile itself.

In addition to the compiled tunnel gravity measurements, we have collected new relative gravity data at the surface, along the tunnel track, to serve as a complementary dataset in constraining the models. We present the new gravity data we collected.

As 2D forward gravity modeling requires density—therefore geological—units and gravity data points to be defined along the same straight line, we also present the processing algorithms we developed to account for the effects of 3D topography in a 2D forward gravity modelling framework (i.e. a 3D-to-2D projection correction), in an internally consistent way.

The main element of this is a novel, density-dependent gravity terrain-adaptation correction, which is applied to the observed gravity data considering 3D topography prior to the comparison with the synthetics from the 2D geological profile. This approach, together with the removal of the regional Bouguer gravity anomaly trend, allows us to speed up the computational phase (without handling and forward modeling 3D geometries), to account for the effect of larger-scale structures, whose modeling falls beyond the scope of this work (e.g. crustal thickness variations), and to focus directly on corrected gravity observations and on the GBT geological units.

We defined and tested different density models, based on the available and up-to-date rock physical properties in the literature, sampled both in the vicinity of the tunnel and across the broader Alpine region (Zappone & Kissling, 2021). The ultimate question was whether any (or which) of these density models allow to corroborate the geological model without modifying its structure and geometry.

## Geological context

We present the GBT geological profile (Guntli et al., 2016) and explain how it has been digitised and adapted for the subsequent analysis. The digitalization process served for extracting the coordinates of the contours of each geological model unit, and to associate them with a given density for the forward gravity modelling. Prior to the modelling phase, both the reference geological model, and the acquired and the compiled gravity data have been projected onto a straight profile, connecting the southern and the northern tunnel portals.

### Tunnel geological profile

**Fig. 2:**
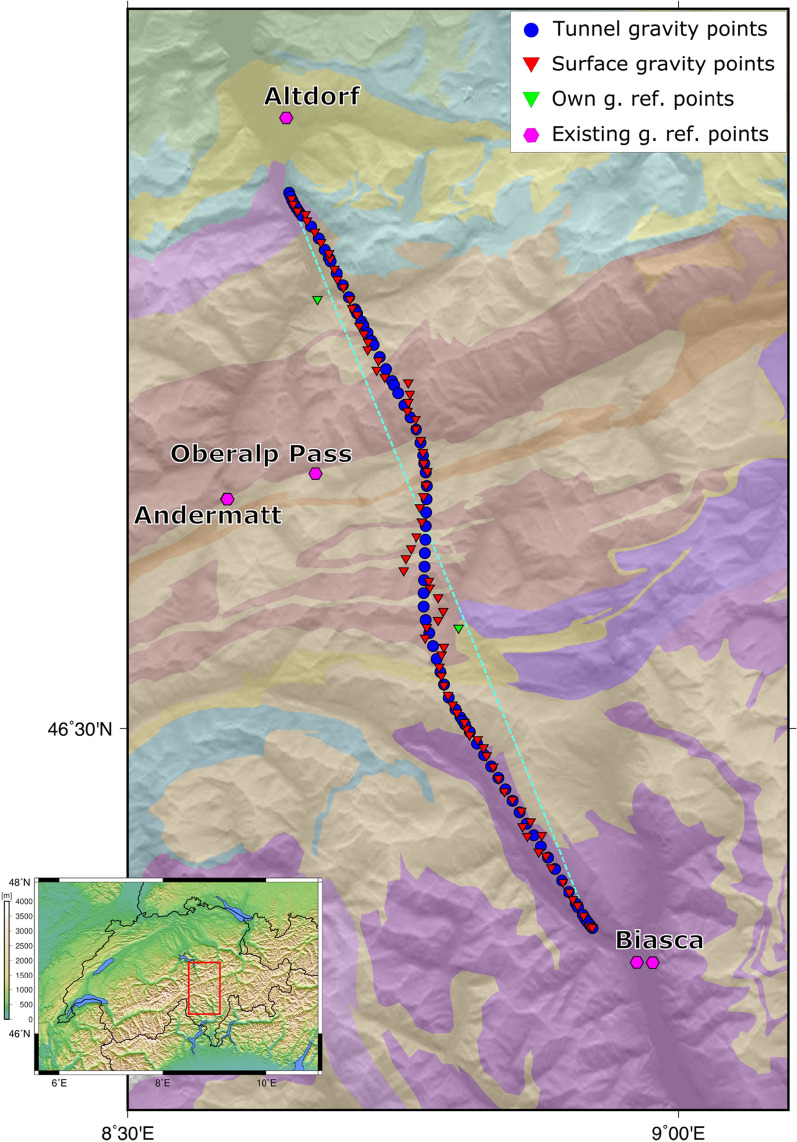
A topographic view of the study area showing the location of the newly acquired 80 gravity points (red triangles) and the compiled tunnel gravity data points (77 points, blue circles). The green triangles represent our newly established relative reference points, which we tied to existing gravity reference points (magenta hexagons) and used to open and close daily measurement loops during the campaigns. The cyan dashed line represents the 2D model reference line, onto which both the geological model units and the gravity measurements are projected for comparison between synthetics and observations using 2D forward modelling. The background colours represent the mapped geological unit geometries at the surface; the geological units are simplified from and freely accessible at https://map.geo.admin.ch/. The inset in the lower left corner indicates the location of the study area (red box) in Switzerland in topographic view

The geological profile along the GBT track has been published in the form of supplementary material to a complete geological, geotechnical and hydrogeological final report on the tunnel (Guntli et al., 2016). The geological profile presents lithological information for the shallow crust along the tunnel track, extending from the surface to 300 m above sea level. It covers the whole length of the tunnel, presenting different rock units of various lithological and geological signature, including an indication for more recent Quaternary sediments (Fig. 1). While the southernmost segment of the GBT geological profile is mainly dominated by gneiss formations with sub-horizontal setting, the central and northern segments present several thin, km-scale units that are characterized by a vertical to sub-vertical structure. These units are intersected by the tunnel profile generally perpendicularly with respect to their strike (Fig. 2), which, together with the surface geological map (Fig. 1), legitimates our 2D modelling approach. The geological profile has been kindly provided by swisstopo in .pdf format, from which we removed all the elements except for the coloured geological units and the reference frame. The reference frame has been set to 0 km horizontal distance at the northern tunnel entrance (which is at 99.727 km in the original document) and to 0 km vertical elevation at the sea-level line, as in the original document. Some of the smallest units have been neglected or merged together. This is the case for small units which are expected to produce a negligible gravity signal and therefore not discernible (e.g. a 120 m wide unit and 1 km in its vertical extent, with a 200 kg $\cdot$ m^−3^ density contrast, would produce a 1.11 mGal gravity signal; a 100 m wide body: 0.97 mGal—compare with modelling uncertainties below) and, similarly, for the tunnel void: a cylindrical tunnel-like cavity with a 9 m diameter (GBT average diameter) would produce up to 0.5 mGal for a measurement point on the floor of the tunnel. The smaller units were most likely interpreted from local observations, either at the surface or inside the tunnel only. Small lenses of limited spatial extent were removed as well. With the use of the legend of the geological profile (Guntli et al., 2016), the remaining formations were labeled and assigned with a lithology. In this way, the simplified along-tunnel 2D geological profile is composed of 69 bedrock geometries, filling the area between the surface and 300 m above sea-level along the GBT.

Steps of this workflow included manual editing in AdobeIllustrator, exporting a .dxf file to be read by QGIS, exporting to .csv format to be read by Matlab, in which we then prepared files compatible with the subsequent 2D gravity forward modelling.

### Compiled density data

Every geological unit of the adapted GBT profile has been assigned with a set of possible density values, from which we built a set density models to test. The density is assumed to be constant and homogeneous within each unit. The main source of rock density information is from rock sample laboratory analyses as provided by the SAPHYR catalog (Zappone & Kissling, 2021). While the latter has been published in the Swiss Journal of Geosciences, the first rock density information was released in 2020 on map.geo.admin.ch from the same source, providing mean density values by lithology, averaged across Switzerland’s broader region.

In the scope of this project, all the available density data in the close vicinity of the GBT was selected from the SAPHYR catalog (121 rock samples in total), and subsequently used to assign density values to each unit based on their location along the profile, and by matching them to the corresponding lithology as reported in Guntli et al. (2016). SAPHYR provides $\rho$_grain_ and $\rho$_wet_ density data: the former was used for samples collected at the surface and the latter for samples collected in the tunnel. Based on the above data, six different density models have been compiled to be tested with gravity data modelling (see descriptions here below and numbers in Table 1).

**Table 1:** Densities in kg $\cdot$ m^−3^ of the main lithologies, for Models 3 to 7 as described in the text

Lithology	Model.3	Model.4	Model.5	Model.6	Model.7
Amphibolite	2861	3006	2648	3180	2700
Dolomite	2885	2540	2081	2885	2700
Gneiss	2710	2687	2587	2790	2700
Granite	2665	2606	2392	2772	2700
Quaternary	2200	1776	1700	2400	2700
Schist	2718	2715	2622	2808	2700
Vulcanite	2650	2641	2531	2716	2700

**Model.1** This model is based on the best guess of density values from SAPHYR point-wise data, by associating to each unit the spatially nearby samples’ density values, considering the same lithology from the SAPHYR database and on the GBT profile. The Quaternary units were associated with 2200 kg $\cdot$ m^−3^. The bodies for which there were no available samples nearby were associated with average values for the same lithology from the 121 selected SAPHYR samples, including sub-specifications for gneiss based on the number of samples and mineralogical composition: 2665 for granite, 2718 for schist, 2974 for amphibolite, 2710 for gneiss, 2664 for two-mica-gneiss, 2755 biotite-gneiss, 2775 for garnet-two-mica-gneiss-schist (all values in kg $\cdot$ m^−3^).

**Model.2** This model is based on the same lithology-wise averages for all the units, with sub-groups for gneiss, and further values such as: 2650 kg $\cdot$ m^−3^ for pyroclastic rocks, 2751 kg $\cdot$ m^−3^ for phyllite and 2740 kg $\cdot$ m^−3^ for paragneiss.

**Model.3** This model is composed of single values for each lithology, without any subgroup for gneiss but using averages from the selected 121 SAPHYR values: 7 different values are used to assign the entire model.

**Model.4** This model is similar to Model 3, but uses the average values of the entire SAPHYR database for the broader region of Switzerland.

**Model.5, Model.6** These models are set to lower and upper end-member density value bounds for each lithology, based on the lowest and highest SAPHYR data-points and the 5th and 95th percentile of regional density distributions per lithology.

**Model.7** In addition to models with rock physics based density data, this model with constant density value of 2700 kg $\cdot$ m^−3^ for all units has been tested.

In summary, the first 6 models range from spatially more variable and locally representative models to more simple models. For all the tested density models, the far-field has been assigned with a constant value of 2670 kg $\cdot$ m^−3^, the same as the area between sea-level and the 300 m elevation line (which is not covered by the GBT geological model). This last addition allows for consistency between the 2D forward modelling and the corrected gravity observations (described in Sect. 4.2.1), requiring no shift at all when comparing the synthetics with the data (Sect. 4.2.3).

### Digital elevation models

We used the *swissALTI3D* digital elevation model provided by swisstopo (see https://www.swisstopo.admin.ch/en/geodata/height/alti3d.html for enquiry and data access) to sample the topography in the vicinity of the surface gravity measurements and along the tunnel line. *swissALTI3D* provides elevation information on a regular grid of 10 m horizontal resolution, compiled in km^2^ tiles. The tiles were merged with the open-source software QGIS, which was also used to sample the digital elevation model at the points of the processing mesh (described in Sect. 4.2.2). Similarly, the SRTM digital elevation model (Farr et al., 2007) was used to sample the topography farther away from the tunnel area and outside Switzerland’s borders, as it provides topographic information at 3 arc-seconds (~ 90 m) resolution.

## Gravity data

We compiled a new gravity database for this study, by merging existing gravity data in the tunnel with the new gravity data we acquired in the field. The prior gravity dataset consists of measurements taken inside the GBT at the time of its construction, while the new gravity data have been measured by ourselves at the surface, as close as reasonably possible to the tunnel track (Fig. 2). The aim of the design of the surface field campaigns was to mimic as much as possible the spatial distribution of the prior tunnel points, which is approximately 1 point every 800 m distance.

This data distribution allows a consistent comparison between tunnel and surface data and also allows to model the density of the relatively smaller units mapped along the GBT geological profile: the median unit horizontal extent along the geological profile is 273 m with a mean of 800 m. In the following, the data collection procedure and the pre-processing practices (from raw data to absolute gravity values) are presented.

### Compiled tunnel gravity data

The available gravity data set was measured inside the GBT by the Swiss Federal Office of Topography (swisstopo: https://www.swisstopo.admin.ch) at the time of the tunnel construction, and it was subsequently compiled for this work. The tunnel gravity data set was provided together with absolute gravity value, coordinates and quality information. One point inside the tunnel was discarded as an outlier with respect to the whole data set, and other 5 points were not taken into consideration as they were measured outside the tunnel portals. The final compiled data set consists of 77 gravity data points, distributed along the tunnel with a mean spacing of 745 m and a median spacing of 933 m. Following the tunnel, the gravity data points range from ~ 315 to 550 m in elevation above sea level.

It should be noted that the final gravity data modelling is done in 2D along a linear profile connecting the two tunnel portals, and to which all the data points are projected perpendicularly: the final, projected tunnel data along the model line has 729 m mean spacing and 886 m median spacing, with a mean projection distance of 1389 m from its original location to the model line.

### Field surveys and new gravity data

We carried out two gravity measurement campaigns, which required in the field ca. 10 days in June 2020 and 1 week in September 2020. The local topography ranges from ~ 300 m above sea level in the valley bottoms to ~ 3000 m at the local summits: substantial physical efforts were required to measure gravity data points at the surface while remaining as close as possible to the tunnel line, and avoiding Quaternary units as much as possible. Each gravity campaign required at least two people in the field, to share the weight of the geophysical equipment (ca. 20–22 kg) and combine driving and planning. We reached some of the points by road in a vehicle and the remaining on foot, with ca. 1000 m vertical elevation change on several hiking days. On two occasions, we benefitted from a one-way helicopter lift to reach the most remote areas and realize physically feasible measurement sessions throughout the day on the way back. At the end of the two campaigns, we collected 80 new relative gravity points, well distributed both in terms of distance from the tunnel and in terms of spacing compared to the tunnel data points (Fig. 2). The mean distance from the surface gravity measurements to the tunnel track is 258 m, while the mean spacing of the surface gravity data points after projection along the 2D model line is 694 m.

All the measurements were organized into daily loops, with each day or half-day of the field campaign always starting and finishing at a location with known absolute gravity value, and mostly the exact same location. This practice allows to estimate the inherent instrumental drift of the Scintrex CG-5 relative gravimeter, which measures the relative gravity difference between the current measuring point and the previous one. Once the instrumental drift is estimated, under the assumption of a linear-trending drift in time over a working day, all the gravity measurements of the same loop are corrected proportionally to the elapsed time. By repeating this approach for every day of campaign, we obtain a relative gravity network, where the relative differences between each point of the network are established, and which has to be tied to a known, absolute reference point to obtain absolute gravity values. This has been done by directly connecting our network of relative gravity measurements to already-established reference points maintained by swisstopo, at Altdorf, Andermatt, Oberalp Pass and Biasca. Furthermore, we have also installed two project-specific gravity reference points with fixed markers in strategic places, one in Amsteg and one at the Lukmanier Pass, for convenient daily loop organization. Ultimately, the entire new gravity dataset is linked to the same reference system as the gravity points in the tunnel. The newly measured gravity data can be made available upon request to the authors.

### Elevation estimate and gravity pre-preprocessing

For each gravity point, the elevation estimate at the measurement site is of fundamental importance for the subsequent data processing and modelling, as each meter of vertical change in the free atmosphere corresponds to a gravity change of $\sim$ 0.3086 mGal. As for other campaigns in the Alps (e.g. Scarponi et al., 2020), a stand-alone GNSS (Global Navigation Satellite System) TopCon receiver antenna has been used to measure the elevation at each site. The satellite signal was recorded for $\sim$ 20 min at 1 Hz sampling rate and the ellipsoidal height was obtained via the PPP (Precise Point Positioning) free processing tool from Natural Resources Canada (https://webapp.geod.nrcan.gc.ca/geod/tools-outils/ppp.php). The PPP processing tool provides processed coordinates in the WGS84 reference frame with ellipsoidal heights, with vertical uncertainties typically of 0.5 m for our acquisition habits (i.e., satellite signal recorded for 15 to 20 min at each location, usually seeing between 6 and 12 satellites), hence corresponding to 0.15 mGal uncertainty on the gravity values. To obtain physical heights, consistent with the local digital elevation model *swissAlti3D*, we used the online tool REFRAME from swisstopo (https://www.swisstopo.admin.ch/en/maps-data-online/calculation-services/reframe.html), which converts WGS84 data to the Swiss Cartesian reference system (LV95) with the physical height system (LN02). Finally, the mean difference between our final elevation measurements and the *swissAlti3D* digital elevation model at the same location is 0.73 m with a median of 0.35 m, which is satisfactory.

The raw gravity data, organized into ordered daily loops, was processed with the GRAVPROCESS software (Cattin et al., 2015) to obtain absolute gravity values. The software allows for proper averaging of the repeated gravity measurements at each site, and removes the time-dependent gravity effect of tides, to finally connect the relative network to the absolute reference points. The mean standard deviation associated with the gravity measurements is 0.063 mGal while the instrumental daily drift was always below 0.1 mGal. Free-air correction and latitude corrections were also computed and applied as described in Sect. 4.2.1. GRAVPROCESS also allows to compute Bouguer gravity anomalies in case one wants to investigate anomalous gravity signal potentially generated by both crustal sources below sea-level and density heterogeneities within the surface topography. As in this study we investigate the gravity effect associated with lithological structures between the surface and the sea-level only, we chose not to compute the Bouguer gravity anomaly but we have adapted the same GRAVPROCESS routines for the direct gravity modelling as described below. Nevertheless, we do account for the gravity effect of crustal thickness variations, by removing the regional Bouguer anomaly trend from the measured data (see subchapter 4.2.1).

### Projection to the 2D model profile

In order to work consistently in a 2D frame, both for the 2D forward gravity modelling and the geological units digitized from the GBT profile, the digitized geological units had to be projected onto a straight line, which we defined as the connection between the tunnel’s northern and southern portals (coordinates are shown in Table 2). From now on, we will refer to this straight projection line as the “reference model line”, which corresponds to the cyan line in Fig. 2.

**Table 2:** Location of the GBT southern and northern portals, which have been used to define the start and the end point of the reference model line and the projections

Portal	Lon. E [°]	Lat. N [°]	Y(LV95) [m]	X(LV95) [m]
North	8.64641	46.83597	2′692′137	1′187′912
South	8.92568	46.37252	2′714′406	1′136′768

Degrees are in WGS84

**Fig. 3:**
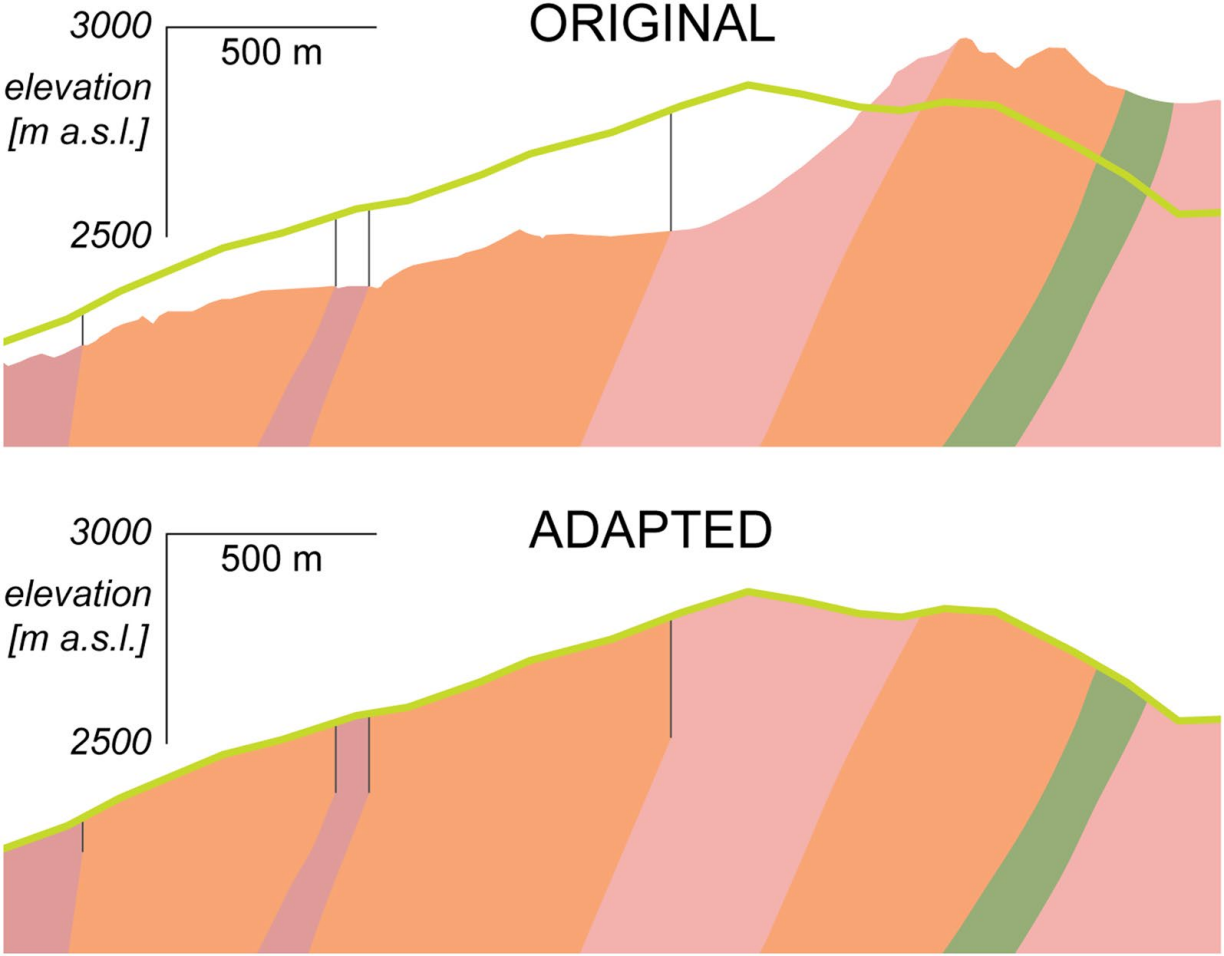
Adaptation of the original GBT geological model profile to the reference model line’s topography (green line). One of the following two steps is performed. If the target topography is higher than the original, lithological boundaries are vertically upward continued (thin black lines), and the geological units (coloured) extended and “filled” with the same lithology (left side of the sample section). In the opposite case, the units are simply truncated at the target topography (right side of the sample section). This example is shown without vertical exaggeration, above 2000 m elevation, and is located at the centre of the profile; the highest peak in the original profile is near Piz Vatgira (2982 m), the highest peak along the reference model line is near the 2888 m peak west of Piz Gannaretsch

The digitized geological units have been projected perpendicularly to the reference model profile connecting the two portals. The mean projection distance is 1486 m with median 1413 m, while the final straight-line distance between the two tunnel portals is 55.783 km. In this phase, an important model adaptation step was implemented. The projected geological units have also been adjusted to the reference model line topography. Consequently, the initial geological model units were either truncated to adapt to the lower elevation reference model line topography, or vertically upwards extended from the unit boundaries until the reference model line topography (Fig. 3).

**Fig. 4:**
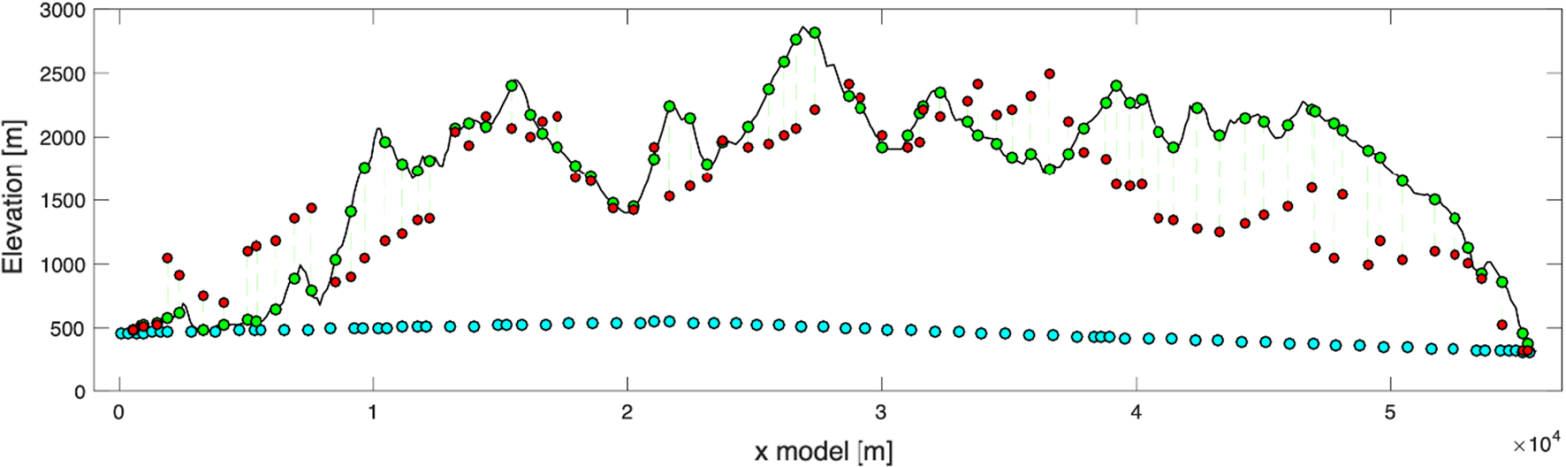
2D topographic profile along the reference model line (see location in Fig. 2), showing the projected tunnel gravity data points (cyan circles) and the projected surface gravity data points (green circles). The surface gravity measurements points at their original measurement elevation are shown as red circles. Prior to comparison with synthetic values, computed at the projected locations (green circles), the gravity data points are corrected for the effects due to this projection. The procedure in explained in detail in Sect. 3

This operation was carried out manually for the initial geological units, to match the reference model line’s topographic profile which is subsequently modelled with gravimetry. This procedure finally produced 62 bedrock units and 2 Quaternary units in total. While these model geometry adaptations may seem to be venturesome choices, the gravity data processing followed the same logic, in order to bring all surface and tunnel gravity points onto the model profile, both horizontally and vertically (Fig. 4). The corresponding gravity processing steps and effects due to this required projection are detailed below, and accounted for and discussed in Sect. 4.2.3.

## Method

In gravimetry, and in geophysics in general, gravity observations are used as an indirect—yet valuable—source of information on the physical properties of the subsurface (e.g. rock density distribution). Within this chapter, we introduce all the technical elements, which we used to evaluate how well a given density model (among other candidates) compares to the gravity observations.

To do so, and for any given density model, we computed numerically the expected gravity signal (also referred to as “synthetic”), which would be produced if a certain density model corresponded to reality, and then compared the so-obtained synthetics with the observations, to assess the density model’s plausibility.

Section 4.1 presents the synthetic gravity computation tools we employed; Sect. 4.2.1 the pre-processing corrections we applied to the gravity observations to remove undesired and known effects; 4.2.2 the topographical data sampled for computing the corrections; 4.2.3 the novel correction term we designed to carry out this analysis in 2D, in an internally consistent manner (i.e. to consistently compare straight-line-projected observations with 2D synthetics).

### 2D gravimetric forward modelling

Forward gravity modelling consists of computing the gravitational attraction effect of a given density distribution in space at a certain point, as a function of the point’s respective distance from the sources and of their densities. In this work, we aim at testing the gravity effect of 2D lithological units of various density, defined based on the 2D GBT geological model. Therefore, the forward problem computation can be substantially simplified with respect to the general three-dimensional case by employing equations assuming a 2.5D structure, with the geometry extending unvaried along the third spatial coordinate (perpendicular to the profile). Under this assumption, each lithological unit can be described as a closed two-dimensional polygon of homogeneous density.

It has been shown in the literature that the gravitational attraction of a two-dimensional body can be expressed as a line integral along its perimeter (Hubbert, 1948). This was later implemented by various authors, providing explicit formulas for the computation of the horizontal and vertical gravity components due to a N-sided polygon of constant density (Talwani et al., 1959; Won & Bevis, 1987). These formulas have been implemented in a home-grown MATLAB software called GRANOM (Hetényi, 2007), which allows to compute gravitational effects both outside and inside a given spatial density distribution, therefore providing in a straightforward manner the synthetic gravity data both at the surface and inside the tunnel for our case study. Eventually, the horizontal and vertical components of the gravity anomaly for an N-sided two-dimensional polygon of given density can be written as:

1 $$\Delta {g}_{z}=2G\rho \sum_{i=1}^{n}{Z}_{i}$$

and

2 $$\Delta {g}_{x}=2G\rho \sum_{i=1}^{n}{X}_{i}$$

where G is the gravitational constant, $\rho$ the polygonal element’s density, Z_i_ and X_i_ the line.

integrals associated with the i-th side of the polygon. The reader is referred to (Won & Bevis, 1987) for the explicit formulation of Z_i_ and X_i_, which represent purely geometrical factors determined by the relative location of the polygon vertices with respect to the point at which the gravitational attraction is computed. We here highlight that the linearity of the gravity field with respect to density of any body, provided its geometry is kept fixed, constitutes a remarkable advantage in terms of computational resources which we will greatly exploit. In fact, it allows to compute once the gravity effect of a given body geometry (Z_i_ and X_i_), and then to scale it linearly with any value of density without additional computational burden. This is true also in the general, three-dimensional case, and will turn out to be a key advantage for computing the 3D density-dependent terrain-adaptation corrections as described in the next section.

As the field observations only measured the vertical component of gravity, the computations of the subsequent model tests were performed for $\Delta {g}_{z}$.

### Testing a density model

In this section, we present the procedure we implemented and ran to test a given GBT reference model with assigned density values, by means of comparison between field gravity observations and the associated forward gravity modelling in 2D. To this purpose, two main points had to be addressed:

1. How to correct field gravity observations, which are sensitive to a complex 3D topography together with subsurface structures, prior to comparison with synthetics associated with a shallow 2D geological density model;
2. How to account for the prescribed GBT test density model in this correction.

Section 4.2.1 describes how the measured absolute gravity data is pre-processed prior to the projection from the 3D *world* to the 2D *model* for comparison with synthetics; Sect. 4.2.2 describes the numerical mesh we defined to sample the topography for the terrain-adaptation correction calculations, and finally Sect. 4.2.3 describes the new terrain-adaptation correction, incorporating consistently the gravity measurements with respect to the 2D reference model topography, that we defined and applied, and coined Terrain-Adaptation Correction (TAC).

#### Classical gravity corrections

The field gravity observations can classically be corrected for a number of factors prior to be corrected for the topography effect and compared to the synthetics obtained from a 2D model. As the final purpose here is to test a density model distribution spanning from the surface to 300 m above sea-level, it is not in our interest to compute a Bouguer gravity anomaly (which would remove the whole effect of the topographic masses with the constant density of 2670 kg $\cdot$ m^−3^) and subsequently re-model crustal and upper-crustal differential densities, but only to account for the difference between the 3D real world and the 2D topographic model derived from the 2D GBT profile (already projected along the 2D model line, as explained in Sect. 3.4).

**Fig. 5:**
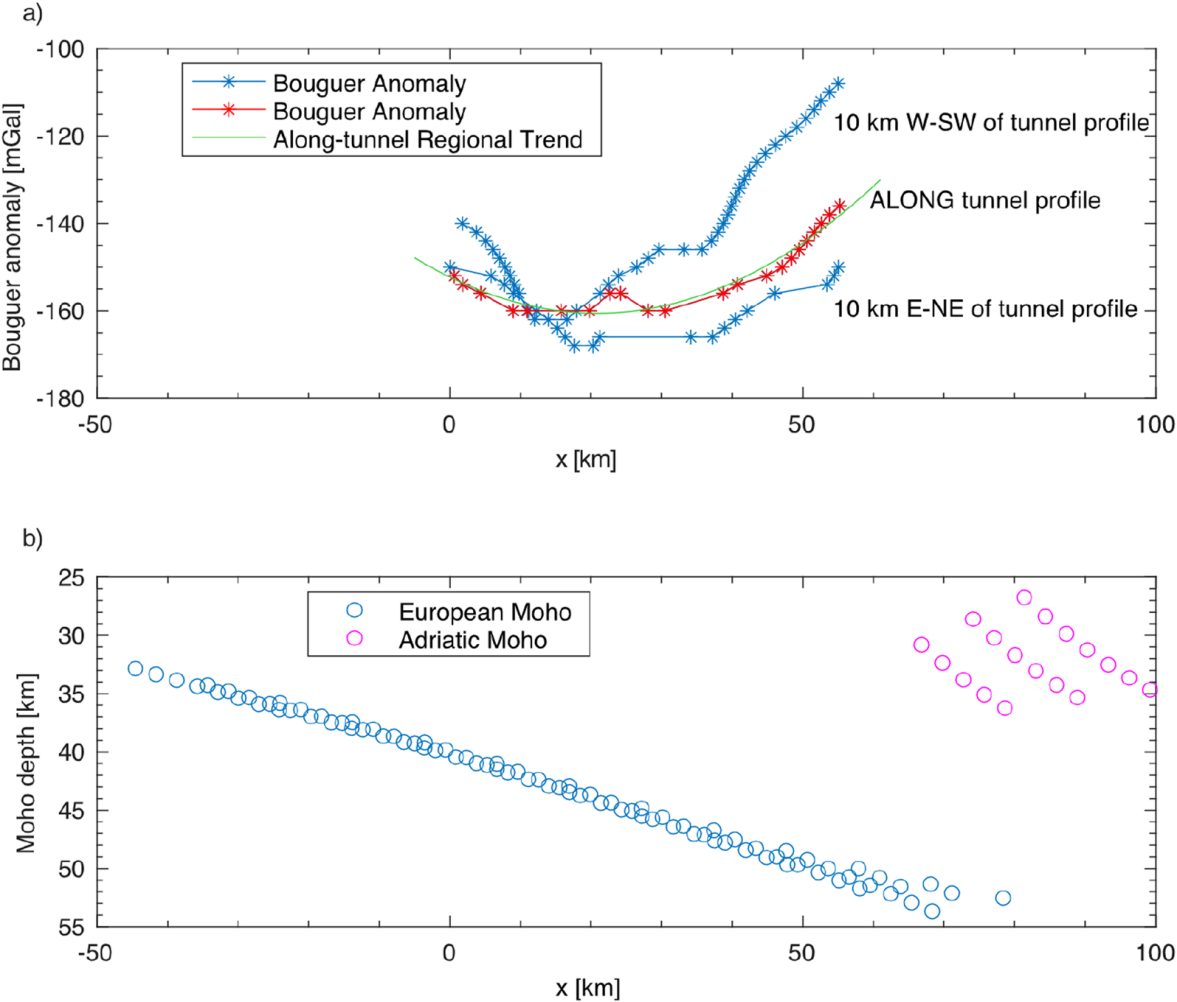
**a** Bouguer gravity anomaly field sampled along the tunnel profile (red), and along two parallel profiles to the W-SW and to the E-NE of the tunnel profile, here reported for comparison (blue). The green line represents the regional Bouguer anomaly trend, interpolated with a 2^nd^ order polynomial and removed from the data to account for the large-scale crustal thickness variations, as shown in panel **b**. The Bouguer anomaly data shown here is provided by swisstopo, accessible at map.geo.admin.ch with layer name “Bouguer anomalies 500” (i.e. Bouguer gravity anomaly map of Switzerland 1:500,000). **b** European and Adriatic Moho depths sampled along the tunnel profile, and along two parallel profiles to the W-SW and to the E-NE of the tunnel profile, as in panel **a**. Moho depth information from Spada et al. (2013)

Therefore, the observed absolute gravity data have been corrected for the following factors:

- **g**_**Lat**_** latitude correction**: this correction accounts for the Earth reference gravity field g_0_ $\sim$ 9.78 m $\cdot$ s^−2^, associated with the reference 0 m elevation at sea-level, especially its variation with latitude, due to the Earth ellipticity;
- **g**_**FA**_** free-air correction**: this correction accounts for the measurement point’s elevation with respect to sea-level and partially balances the latitude correction by taking into account elevation: for every meter of vertical upward displacement in the free atmosphere the reference gravity field decreases by 0.3086 mGal;
- **g**_**BAtr**_** regional trend correction**: this correction accounts for the regional trend of the Bouguer gravity anomaly field, available from the regional map (1:500′000 scale, 2 mGal contour line) of swisstopo. We consider that the regional field accounts for crustal changes below sea-level, and in our region it is mainly shaped by the major Moho depth change across the Europe-Adria plate boundary (Fig. 5b). For this step, the regional Bouguer trend was sampled along the tunnel profile and interpolated with a 2^nd^ order polynomial. The model profile crosses the most negative parts of the regional Bouguer anomaly map, with a flat-plate shape along the profile and relative amplitude changes on the order of 10 mGal (Fig. 5a). Two additional regional trends calculated along two profiles to the east and west of our line corroborate the shape and amplitude of this correction (Fig. 5).

Removing the regional Bouguer gravity anomaly trend from the observations (g_BAtr_) allows to account for the larger-scale gravity effect of crustal thickness variations, and to model directly the so-corrected absolute gravity values, in relation to the shallow geological units along the GBT profile.

#### Numerical mesh for sampling topography

**Fig. 6:**
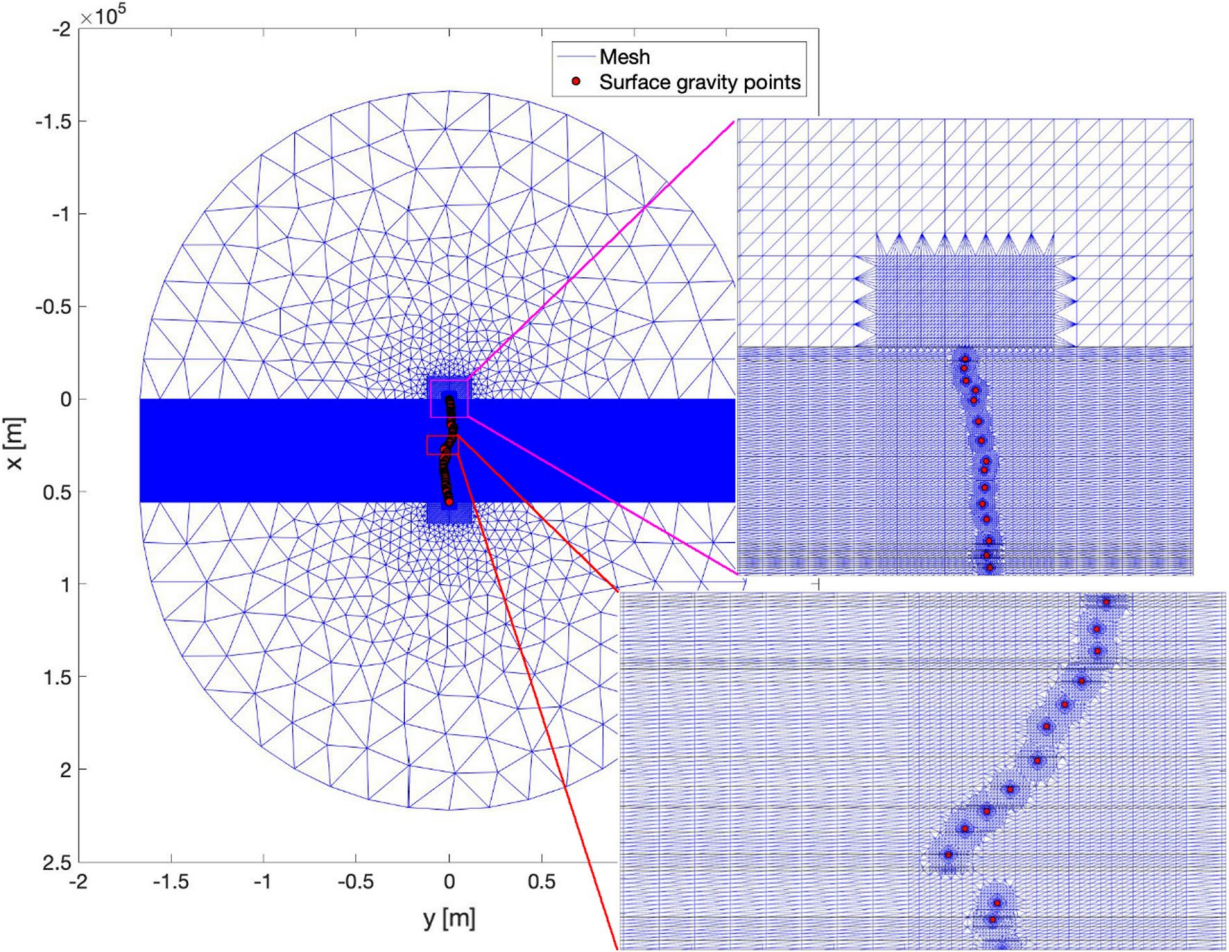
Graphical representation of the numerical mesh used for the density-dependent gravity correction computation. The digital elevation model (either swissALTI3D or SRTM) depending on the required resolution) is sampled at each mesh node and subsequently used for the computations. The resolution of this static mesh is higher (10 m) in the vicinity of the gravity measurement points (red circles) and along the reference model line (including the original GBT track), while it decreases with increasing distance from this central zone. The two insets show the varying spatial resolution for two particular areas, along the reference model line and at around the northern tunnel portal. The thin horizontal black lines in the two insets mark the boundaries between geological units at the surface digitised from the GBT geological profile, which were included as a line of nodes in the mesh. It is of fundamental importance that the mesh respects these boundaries, allowing for a correct and consistent density mapping from the 3D structures to the 2D profile, for the computation of the terrain-adaptation corrections

A proper evaluation of the gravity effect of the 3D topography is of primary importance for the subsequent density-dependent gravity corrections, required to project the field gravity measurements to the 2.5 structure defined by the GBT profile. The numerical mesh used in the computation is an ensemble of points over which the digital elevation model (either *swissALTI3D* or SRTM, depending on the required resolution) is interpolated. A digital elevation model is usually delivered on a regular grid, which is not directly suitable for the numerical computation of the desired gravity corrections here, as it would require unrealistically large computational power. Furthermore, as the effect of gravity decreases with inverse of the square of the distance, a constant resolution is not required across the whole study area. While other authors in similar contexts defined a mesh of dynamically decreasing resolution for each processed gravity point (e.g. Cattin et al., 2015), for this application we defined one dedicated, static mesh with several zones of spatial node density (Fig. 6). The mesh is denser around each gravity point and along the model and the tunnel lines, while its resolution decreases with distance away from this area. Most importantly for the subsequent testing of different density models via gravity modelling, the mesh includes lines of nodes representing the surface projections of all considered geological unit boundaries, as prescribed from the adapted GBT geological profile. This allows to properly compute the effect of each 2.5D geological model unit’s structure. The geometries of these units are kept unchanged until they are proven unsatisfactory. Such a consideration of lithological units allows to evaluate the effect of various density models easily via simple linear scaling.

#### 3D-to-2D density-dependent terrain-adaptation correction

The most critical and novel correction to apply here is a density-dependent terrain-adaptation correction and simultaneous topography-adaptation prior to comparing the observations with the synthetics, obtained for the 2D GBT density model. This correction accounts for the gravity effect due to the difference between the 3D *real-world* topography, where the gravity measurements have been taken, and the 2.5D *model* structure that the forward gravity modelling accounts for (as the synthetic gravity signals are computed along the reference model’s topography, and not at the original gravity measurement points).

This 3D-to-2D projection correction is computed independently at each gravity measurement location and for each geological unit. Each of the geological units is treated as a single 3D rock volume bounded by a top surface (respectively the 3D or the 2.5D topography depending on the term in Eq. 3), a flat bottom and vertical sidewalls. The units’ vertical sidewalls, or lateral boundaries, have been defined by extending the surface expression of the 2D geological units perpendicularly to the profile line. Initially, a constant density of 1000 kg $\cdot$ m^−3^ is used as reference value for the computation of the single correction terms related to each gravity-point-geological-unit couple. Eventually, the correction terms—for each geological unit—are linearly scaled according to the current density model and summed up at each gravity location. This means that the geometrical factor of gravity contributions was computed only once in this study for a given mesh, saving considerable time.

**Fig. 7:**
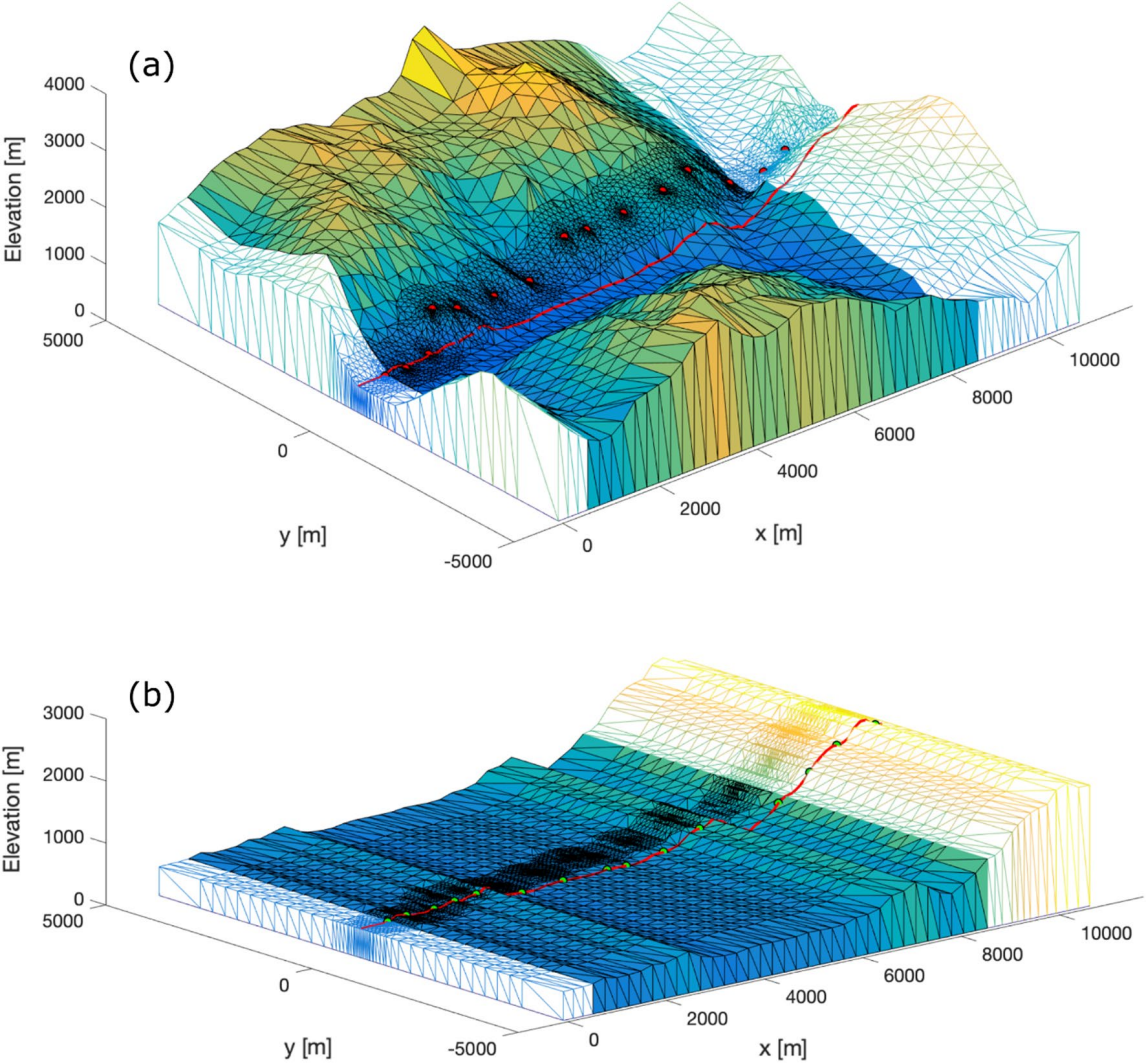
**a** Initial real-world situation at the northern tunnel portal. The 3D topography is interpolated at mesh nodes from the *swissALTI3D* DEM. The topographic mesh coloured by elevation is associated with a single unit as indicated by the 2D GBT geological profile, while the neighbouring units are shown with coloured mesh edges only. The mesh resolution is higher in the vicinity of the surface gravity measurement points (red circles) and in the vicinity of the reference model line (red line), which is by definition at y = 0 m along the whole study area. The topography sampled along the reference model line corresponds to the reference model topographic profile, and it is the same profile to which the geological profile was adapted, and which is used for the synthetic gravity computation. **b** Final, adapted reference model situation of the same area at the northern tunnel portal. View of the 2.5D topography as laterally continued from the 2D reference model line topographic profile (red line). This 2.5D structure is correctly accounted for by our forward gravity modelling routine, which neglects any topography variation along the profile-perpendicular coordinate (Won & Bevis, 1987). The red line following the topographic profile at y = 0 m represents the 2D reference model line, to which the observed gravity measurements are translated (green circles) prior to the comparison with the synthetic values. The newly introduced terrain-adaptation correction (TAC, see Sect. 4.2.3) takes care of all changes from **a** to **b**. It should be noted that the spatial extent of each unit block guarantees the consideration of topographical masses for a minimum radius of 167 km around each gravity measurement, and that horizontal axes in panels **a** and **b** were limited for displaying purposes

Therefore, for each gravity point, the density-dependent terrain-adaptation correction (TAC) is defined as:

3 $${g}_{TAC}=\sum_{j=1}^{N}{\rho }_{j}\cdot ({TMC}_{2.5D}^{f}-{TMC}_{3D}^{i})$$

where N is the total number of geological units, each of which is assigned with a density value $\rho$_j_ prescribed by the 2D geological model, TMC^i^_3D_ (Topographical Masses Contribution) is the gravity effect of the topographical masses computed for the original 3D topography at the ***initial*** real-world gravity measurement point location (Fig. 7a), and, similarly, TMC^f^_2.5D_ is the gravity effect of the topographical masses computed for the 2.5D structure defined at the ***final***, projected points, located along the 2D reference model’s topography line (Fig. 7b). With this approach, the classical terrain correction and the translation of observations points to the reference model profile, including adaptation to the new model topography (Fig. 7), are all realized in the same step, and they all consider density-dependent lithological units.

The MATLAB code at the core of the computation of the gravitational effect, due to a 3D rock volume of homogeneous density, was imported and adapted from the software GRAVPROCESS (Cattin et al., 2015). The summation over the unit-wise gravity contribution terms was then tested, in a planar setting with flat topography and no relief, against the theoretical Bouguer plate correction formula (δgB = 2πGρh, Turcotte & Schubert, 2002), yielding the correct result well below 1% error.

The final, observed gravity product, ready for the comparison with the synthetic gravity values, is defined as follows:

4 $${g}_{corr}^{obs}={g}_{meas}^{obs}-{g}_{Lat}+{g}_{FA}-{g}_{BAtr}+{g}_{TAC}$$

where ${g}_{meas}^{obs}$ is the absolute gravity value obtained from field measurements and classical pre-processing (as described in Sect. 3), g_Lat_, g_FA_ and g_BAtr_ are the latitude, free-air and regional Bouger-trend corrections described in Sect. 4.2.1, and g_TAC_ is the newly introduced density-dependent terrain-adaptation correction for the translation of surface gravity measurement and adaptation to the 2D reference topographic profile. The same procedure is applied both for the surface and the tunnel gravity measurement points, with the only difference that the tunnel points are projected along the 2D model line keeping their initial elevation. We emphasize that as long as model unit geometries are kept fixed, the TAC is linearly scalable with density, therefore the unit densities in the TAC are consistently always the same as in the synthetic forward model at practically no additional computational cost.

## Results

The density models proposed above (Sect. 2.2) based on rock physics data have been tested by comparing the associated 2D forward gravity model’s signal with the gravity observations corrected using the same density models (Eq. 4). Each density model has been tested separately, for both the surface and the tunnel gravity datasets, and their respective performance in terms of fit of the synthetics to the observations has been quantified by computing the root-mean-square (RMS) error:

5 $${RMS}_{model}^{surf, tunnel}=\sqrt{\sum_{i=1}^{N}\frac{{({syn}_{i}^{surf, tunnel}-{obs}_{i}^{surf, tunnel})}^{2}}{N}}$$

where N is the number of data points running with index i, and superscripts *surf* and *tunnel* represent the surface and the tunnel points. The obtained misfits for the tested density models are shown in Table 3. We emphasize that no shift of the synthetic gravity profile was required, with all the assumptions made above the synthetic values fall very close to the observed ones.

**Table 3:** Root-mean-square misfit obtained by comparing synthetic forward modelling results and corrected gravity observations, for both surface and tunnel data, for various density models

Density model	Surface RMS [mGal]	Tunnel RMS [mGal]	Total RMS [mGal]
Model 1	2.83	3.85	3.37
Model 2	2.80	3.63	3.23
Model 3	2.76	3.81	3.31
Model 4	3.34	3.21	3.79
Model 5	8.82	8.67	8.75
Model 6	4.44	4.71	4.57
Model 7	2.66	3.93	3.35

Model 7 is a constant density model for all units ($\rho$=2700 kg $\cdot$ m^−3^). Surface and tunnel data points are given the same weight

**Fig. 8:**
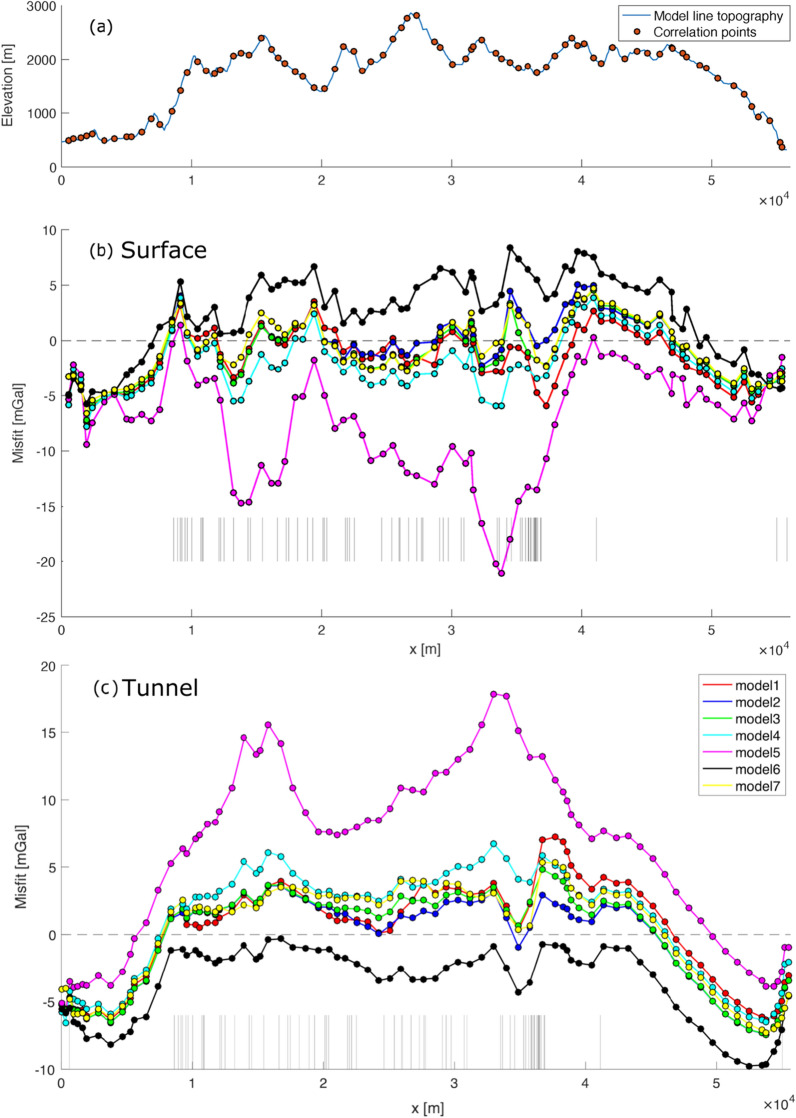
Misfit profiles between modelled and synthetic gravity anomalies, considering different density models. **a** The topographic profile along the reference model line (blue line), together with the projected gravity data points at the surface (orange points). These points have been used to sample the topographic profile and compute the cross-correlation between topography and misfit. **b** Point-wise misfit for the surface gravity data points, for each of the tested density models (see colour legend), as described in Sect. 2.2. Black thin lines in the lower part of the figure, indicate the location of the geological unit boundaries at the surface, along the profile. The point-wise misfit represented in the figure is defined as synthetic minus observed gravity value. **c** Same as **b** for the tunnel points

Along the tested density models, Model 2 seems to yield the best total RMS, however Models 3, 7 and 1 are also very close to it. The misfit is also represented by point-wise difference between synthetic and observed gravity values along the profile, both for surface and tunnel gravity data (Fig. 8b and respectively Fig. 8c). As it can be seen in Table 3, the reported misfits for the tunnel gravity points are slightly higher than those for the surface points. While we expect tunnel points to be less sensitive to topography, they may be more influenced by the geological vertical-unit-boundary assumption used in the TAC computation, and by the lateral extension of the 2D GBT geological profile (see chapter 6.1 for further details).

As the overall shape, the long wavelength pattern of the misfit curves (Fig. 8b and c) seems to reflect the model topography (Fig. 8a), their correlation has been quantified. This has been computed as the zero-shift cross-correlation, according to the formula:

6 $$xcorr\left({\varvec{g}},{\varvec{t}}\right)=\frac{\sum_{i=1}^{N}{g}_{i}\cdot {t}_{i}}{\sqrt{(\sum_{i=1}^{N}{g}_{i}\cdot {g}_{i})\cdot (\sum_{i=1}^{N}{t}_{i}\cdot {t}_{i})}}$$

where ***g*** represents either the surface or the tunnel gravity misfit points and ***t*** represents the topography, sampled at the same x-coordinates as the surface respectively tunnel gravity points along the model line (see locations in Fig. 8a). The results obtained Eq. 6 are shown in Table 4.

**Table 4:** Zero-shift cross-correlation values of the surface and the tunnel gravity misfit with topography for each density model

Density model	Surface–topo xcorr	Tunnel–topo xcorr
Model 1	− 0.340	0.324
Model 2	0.014	0.088
Model 3	− 0.142	0.197
Model 4	− 0.500	0.466
Model 5	− 0.844	0.842
Model 6	0.728	− 0.593
Model 7	− 0.019	0.292

To compute the cross-correlation, the topography along the model line has been sampled as the same x-coordinate as the gravity data, as shown in Fig. 8

Similarly to what is argued for Nettleton’s method (Nettleton, 1939, 1976), that the optimal density for near-surface structures should provide the least correlation between Bouguer anomalies and topography, these cross-correlation values (Table 4) may be interpreted as indicators of how well the local unit densities, used for both the observation corrections and the forward gravity modelling, fit the data and in turn relate to the real-world density structures. Intuitively, models showing no correlation with the topography (cross-correlation closest to 0) can be considered as best performing models, and here again Model 2 shows the top performance, while all other models perform less well. Note that both the RMS and the cross-correlation based misfit evaluation shows differences for the surface and the tunnel data, which strengthens our motivation to use both datasets in future studies.

Ultimately, a systematic inversion for the best-fit density for each unit could be set up, which would be made possible thanks to the stored geometrical factors and straightforward scalability of gravimetric effects with changing density.

### Sensitivity tests

**Fig. 9:**
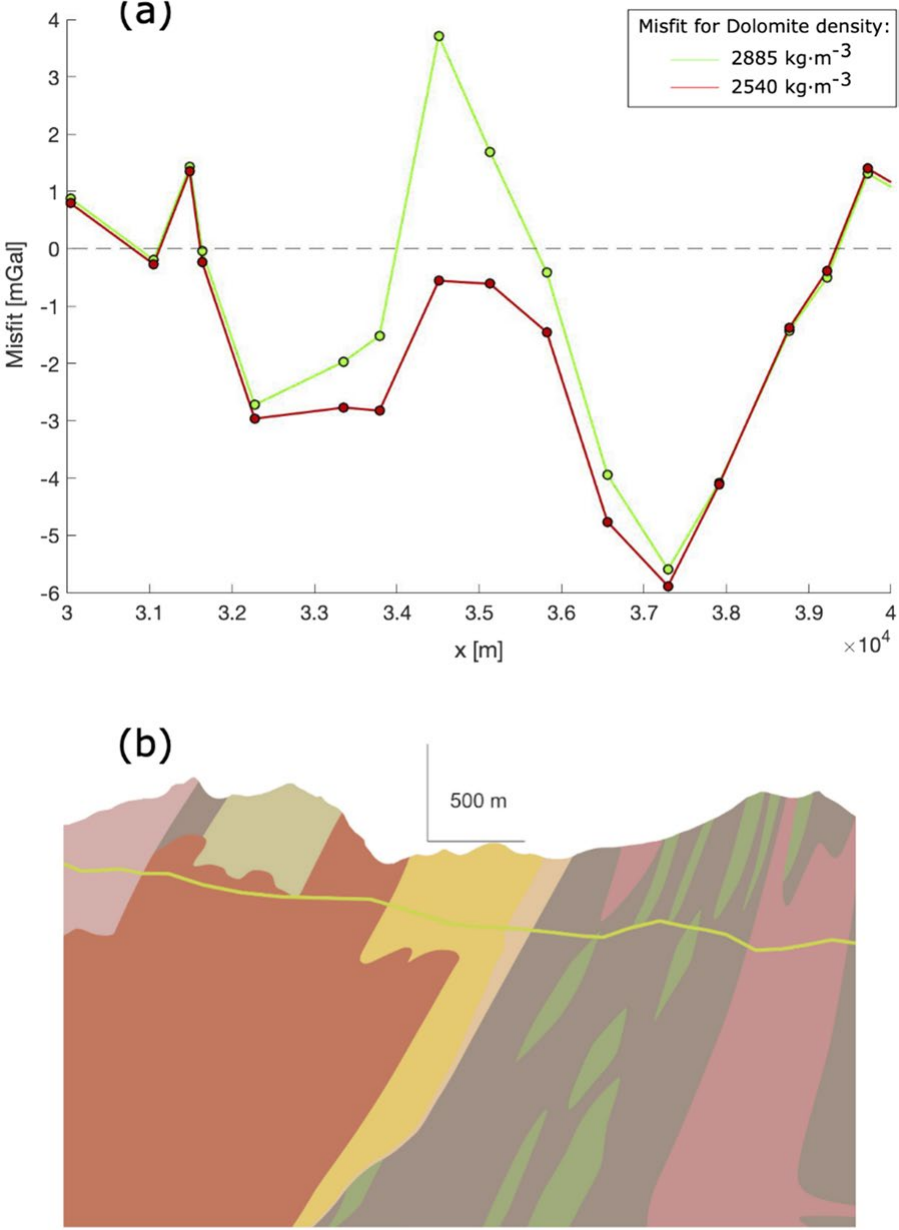
Gravity effect due to a density variation of $\sim$ 300 kg $\cdot$ m^−3^ of a middle-size unit (Piora Dolomite), presenting a $\sim$ 600 m × 400 m topographic adaptation. **a** Gravity misfits, defined as the difference between synthetic values and corrected observations, for a higher density value of 2885 kg $\cdot$ m^−3^ (green line and dots) and for a lower density value of 2540 kg $\cdot$ m^−3^ for the Dolomite unit (yellow unit in panel **b**). The remaining unit densities are unvaried. **b** Geological profile as projected along the straight reference model line. The green line is the reference topography, and separates the modelled geological units below and the original model that includes the parts above. The latter excess is removed and accounted for by the TAC correction in the transition from the 3D to the 2.5D model

The density-dependent terrain-adaptation correction (TAC, Eq. 3) is computed as a sum of unit-wise contributions, which are linearly scaled according to the tested density model. As the unit geometries are heterogeneous and highly variable along the geological model, all the terms summing up to the final TAC vary as a function of the distance from the gravity point and of the density the units are associated with. This is here demonstrated by taking a middle-size example with quite variable density values according to the different models, the Piora Dolomite: the associated density can vary from 2885 kg/m^3^ to 2540 kg/m^3^, depending on weather the single local rock-density value or the lithology-wise SAPHYR catalog average are considered, respectively. The TAC associated with a density variation of $\sim$ 300 kg $\cdot$ m^−3^ for this unit, which features a $\sim$ 600 m × 400 m topographic adaptation from the 3D to the 2.5D model in the vicinity of the model line (Fig. 9b), causes a misfit variation exceeding 4 mGal for the nearest gravity point, gradually tapering down to negligible ca. 2 km away from this particular unit (Fig. 9a). Such a ca. 4.5 mGal signal stands well above the mGal interpretation threshold we identify in this study (see discussion in subchapter 6.1), and the 1 mGal interpretability level allows us to constrain, while keeping everything else constant, the density of this unit at 77 kg/m^3^ precision. In general, the larger the body the better the density constraints obtained from gravity data.

**Fig. 10:**
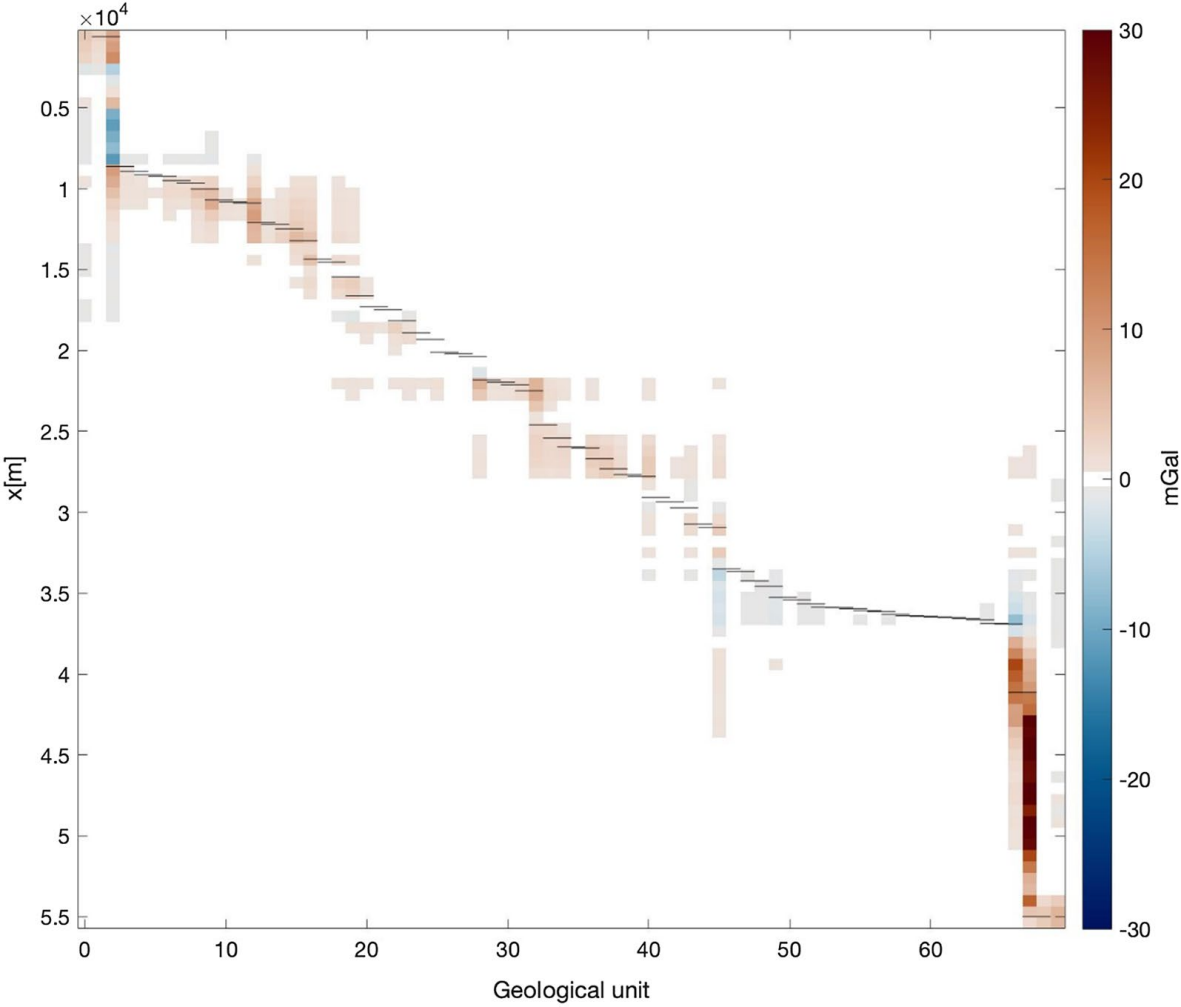
Terrain-adaptation gravity correction due to each geological unit (ranging from North to South on the x-axis) and as a function of the selected surface gravity points (shown at their location along the model line on the y-axis from North (top) to South (bottom)). The gravity values shown here are for a reference density of 1000 kg $\cdot$ m^−3^. The variations depicted here depend on each unit’s geometry and the distance between the unit and the selected gravity point. The mainly diagonal structure indicates that units tend to affect primarily the gravity points close by along the profile, with the exception of the widest units at the beginning and the end of the model line. For given units (i.e. along a single column) some gaps are present between non-zero values, due to the different elevation change between the unit and the gravity points. The thin horizontal lines, plotted for each unit along the y-axis indicate the northern and the southern boundary of the associated unit, hence showing how far beyond its own boundaries a geological unit is affecting the gravity points

A more complete view of the magnitude of the TAC due to each unit and for a reference density of 1000 kg $\cdot$ m^−3^ is shown in Fig. 10. This provides information on each unit’s "geometrical" factor contributing to the TAC, which is mainly determined by the location of the projected gravity points along the 2.5D model line with respect to the surrounding original 3D topography. The widest units provide the strongest contributions, while the smaller units account for little corrections even for the nearby gravity points.

## Discussion

### Data and modelling uncertainties

In this sub-section, data and modelling uncertainties are discussed in two groups. Part of the uncertainties can be quantified via estimation of standard deviations, while other sources of uncertainties are well recognized but more difficultly estimated.

Each gravity point’s elevation measurement is associated with a standard deviation of 0.5 m, which translates into a $\sim$ 0.15 mGal uncertainty on the gravity data. In addition to this, processing uncertainties are also associated with the raw-to-absolute gravity data processing. Standard deviations obtained from the GRAVPROCESS software are indicated in Table 5.

**Table 5:** Mean standard deviations in mGal associated with the gravity data products obtained from GRAVPROCESS during the processing of the raw gravity data acquired in the field

Mean Standard deviation	[mGal]
Absolute gravity values	0.06
Free air correction	0.016
Relative network adjustment	0.053
Absolute network adjustment	0.03

All the effects presented above account for a standard deviation of $\sim$ 0.3 mGal in total.

Furthermore, we systematically tested the effect of the numerical mesh density on the final misfit. By increasingly densifying the computational mesh, we observed that a spatially 25% denser mesh with respect to the original one, produces a ~ 0.3 mGal variation on the TAC and, hence, on the final misfit. Further increasing the mesh density results in smaller and smaller changes on the final misfit.

Other effects are source of uncertainty, but more difficult to quantify, primarily the assumption of vertical geological unit boundaries for the TAC computation. While the geological model features mostly vertical to sub-vertical units, it presents unit boundaries deviating from the vertical as well, and which could not be accounted for in the numerical calculations. Moreover, the tunnel-perpendicular extension of each unit in a straight manner to 2.5D is certainly a simplification (see Fig. 1). The limited vertical extent of the GBT geological model (from surface to 300 m above sea level) may also explain part of the obtained negative misfit trends near the northern and southern tunnel portals, where the gravity points are very close to the 300 m elevation line. A constant density of 2670 kg $\cdot$ m^−3^ was adopted for the rocks between sea-level and 300 m elevation, in order to stick to the published model and to avoid making further assumptions. The effect of light Quaternary sediments is difficult to quantify as well, as their density can be highly variable, and they surely influence the data near the tunnel portals where they exceed 100 m thickness, although only within the valley. Given the level of structural simplifications without unsubstantiated assumptions, it is positively surprising to obtain so well-fitting results.

In general, we do not observe a systematic correlation between the final misfit and the elevation change between the original and the final projected surface point elevations during TAC, showing that the TAC correction is performing well in its 3D-to-2.5D projection purpose. This is particularly valid for the best performing models (Additional file 1: Fig. S1).

In addition to this, a further densification of the numerical mesh at the along-tunnel projected surface points do not cause significant changes in the final misfit near the tunnel portals (Additional file 2: Fig. S2), and the patterns shown in Fig. 8 remain.

Finally, some amount of misfit may stem from simplifying model assumptions such as homogeneous density within the geological units, while, in reality, there may be local density variations due to geological phenomena such as karstification (e.g. Piora unit) or the presence of amphibolite in some of the gneisses (e.g. Lucomagno area).

Overall, and considering the cumulative effect of these aspects, it seems reasonable to interpret from one to a few mGal variations, without addressing sub-mGal features, which seems to be an acceptable level for a model simplified into 2D.

### A quest for the best model?

The results presented in Sect. 5 already provide a base of quantitative information on how the various tested density models compare with respect to the gravity observations. It appears clear from Tables 3 and 4 that Models 5 and 6, which have been defined to represent the lower and upper bounds for each lithology’s density, can really be regarded as end-member models. It is also possible to see that between Models 3 and 4, which have been defined by averaging the density values for the main lithologies respectively from local and regional samples, the local data (Model 3) provide better fit. Including higher spatial variability of densities based on local samples (Models 1 and 2) yield some gain in the fit, Model 2 being numerically the best. Still, Models 1 to 3 present relatively small differences in terms of RMS and cross-correlation value, and their relative differences are close to the uncertainty of the data and the processing. Model 7, with a homogeneous density value of 2700 kg $\cdot$ m^−3^ for all the units, provided the best RMS fit and second best no-topo-correlation result for the surface gravity observations but performed much worse for the tunnel gravity observations.

**Fig. 11:**
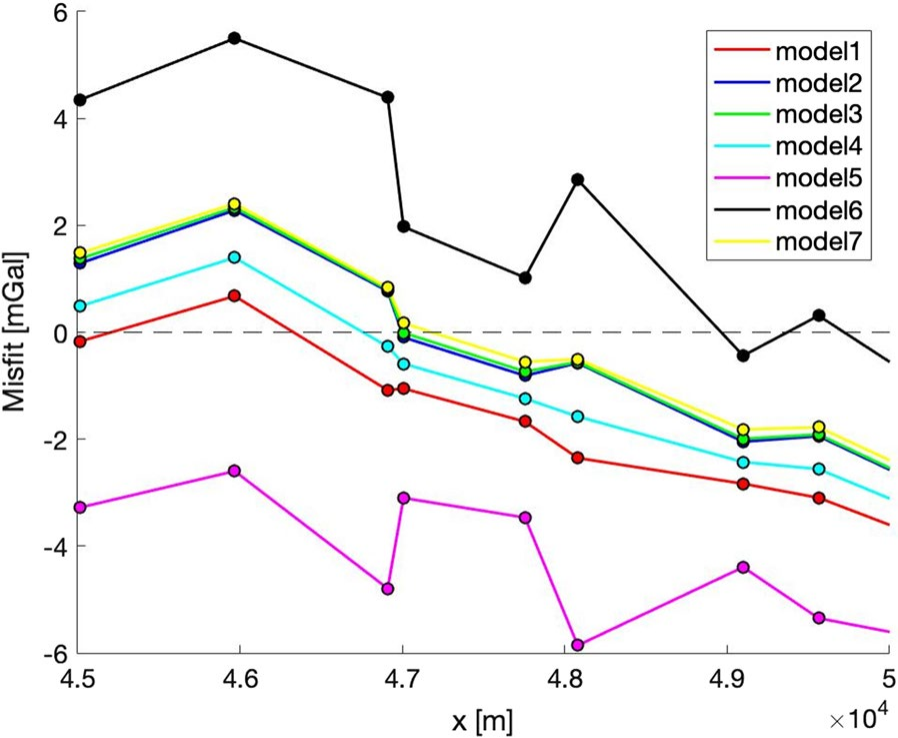
Point-wise misfit for the surface gravity values, with a focus on the 45 to 50 km section along the model line within the same geological unit. Models 5 (low density) and 6 (high density) present short-wavelengths symmetric steps, which strongly correlate with the position of two pairs of surface gravity points at 47 and at 48 km distance acquired at significantly different elevations. These misfit steps do not appear or are smaller for the other density models, which means that measurements taken at different elevations have the potential to constrain local densities. The point-wise misfit represented in the figure is defined as synthetic minus observed gravity value

On one hand, this may suggest that the surface gravity data points are less effective in constraining small-scale subsurface heterogeneities than the tunnel points, the latter being inside the modeled domain. On the other hand, even for surface gravity points, Model 7 is not the best everywhere: by looking at Fig. 11, Model 7 provides a worse fit on the left-hand side of the considered profile section and a higher correlation with the local elevation profile.

Ultimately, Model 7 is obviously too simplistic from a geological-lithological perspective, and its relatively good fit is likely due to its single density value, being representative of a reasonable average value for the whole area.

By looking at the point-wise data misfits (Fig. 8b, c), it is also possible to notice that there is no single density model providing a systematically better misfit along the whole profile. While it appears clear that density contrasts are required to fit both surface and tunnel gravity data sets, and that diverse in situ density information (SAPHYR) provide a better fit to the data, it is not yet possible to state whether one of the tested models should be considered as the best density model for the GBT geological profile. Nevertheless, further elements can be considered to reduce the circle of the well-fitting density models and to improve individual unit’s best density estimates. Focusing on particular units may be of interest, for example by looking at the surface gravity misfit curves at $\sim$ 47.5 km distance, where the GBT geological model presents a gneiss unit. There, Model 5 and Model 6 present short-wavelengths symmetric steps (Fig. 11), which strongly correlate with the original surface gravity point elevations acquired very close to each other but at significantly different elevations. These steps do not appear in the other four, better fitting models, and making the profile as smooth as possible could be set as a target when looking for its density estimate. In this case, we can see that Models 5 and 6 provide suboptimal values for this local gneiss unit (2587 and 2790 kg/m^3^ respectively). On the other hand, while we identify Models 2, 3 and 7 as the locally-best performing models in terms of misfit, we do not further interpret their relative sub-mGal differences, as they also present relative density differences on the order of magnitude of 10 kg/m^3^ for this area. By looking at the surface misfit profile (Fig. 8b), it’s possible to pinpoint several other sections where the density models provide identifiable and different misfits: e.g. Model 4 underperforming at around x = 12 to 18 km and x = 22 to 33 km; Model 7 being separated from the best performing models at x = 15 and 25 km; Model 2 performing better than all the other models at x = 35 km, or again at x = 11 km for the tunnel gravity data points (Fig. 8c).

Finally, we emphasize that a well-sampled case study of the GBT, both in terms of gravity data and in terms of rock densities, provides a satisfactory geophysical validation of the local geological model, and that no structural modification of the originally published GBT geological model (Guntli et al., 2016) was required. In that sense, at the considered spatial scales of sub-km features, the geological interpolation of surface and tunnel data seems to have been reasonable.

## Conclusions

We have collected 80 new gravity data points along the GBT track at the surface, and combined them with 77 existing gravity data points measured inside the tunnel. For the dual purpose of testing the published geological profile and of developing a new gravimetric processing scheme, we adapted the along-tunnel geological profile, associating each geological unit to a lithology with density data from the SAPHYR catalog, and defining a series of density models to test. Both the geological and gravimetric information have been projected and adapted onto a 2D reference model, to consistently perform forward gravity modelling and comparison to the observations. We developed a fully 3D, density-dependent terrain-adaptation correction, as part of the internally consistent 2D model preparation, which is applied prior to comparison between the observations and synthetics, and whose contribution has been quantified for all the considered geological units. The analysis of the gravity data misfits indicates that density information obtained from in situ rock samples provide in general a better fit than average density values from the broader Alpine domain for the same lithologies. The best fitting model, for the ensemble of the surface and tunnel gravity points, presents also the lowest correlation with the model’s topographic profile. Short-wavelengths variations along the gravity misfit curves can be explained as well, and could be further exploited. Nevertheless, data and modelling uncertainties suggest not to interpret sub-mGal variations. Eventually, either for this and/or similar settings, we suggest that a systematic exploration of the possible density models can be performed by setting up and inversion problem framework. Overall, the geological structure proposed during the construction of the GBT can be well fit with the density values available for most of the local lithologies, as verified by our gravimetric analysis.

## Supplementary Information

**Additional file 1.** **Top panel)** 2D reference model topography; elevation of the original surface gravity measurement points (yellow) versus elevation of final 3D-to-2.5D-projected surface gravity measurement points (red). **Bottom panel)** Surface gravity model misfit versus 3D-to-2.5D-projection elevation change (blue thick line). **Table)** Zero-lag crosscorrelation between surface gravity model misfit and elevation difference, surface gravity model misfit and elevation; root-mean-square (RMS) misfit.

**Additional file 2.** Surface gravity model misfit and associated variation (dashed lines) due to a 25% densification of the numerical mesh. These displayed points are located at the northern edge of the tunnel profile.

## Data Availability

The newly measured gravity data can be made available upon request to the authors.
