# Supplementary figures and images for: A gravimetric assessment of the Gotthard Base Tunnel geological model: insights from a novel gravity terrain-adaptation correction and rock physics data
Source: Swiss J Geosci. 2022 Nov 11;115(1):22. doi: 10.1186/s00015-022-00422-z (PMC9652475; doi:10.1186/s00015-022-00422-z)

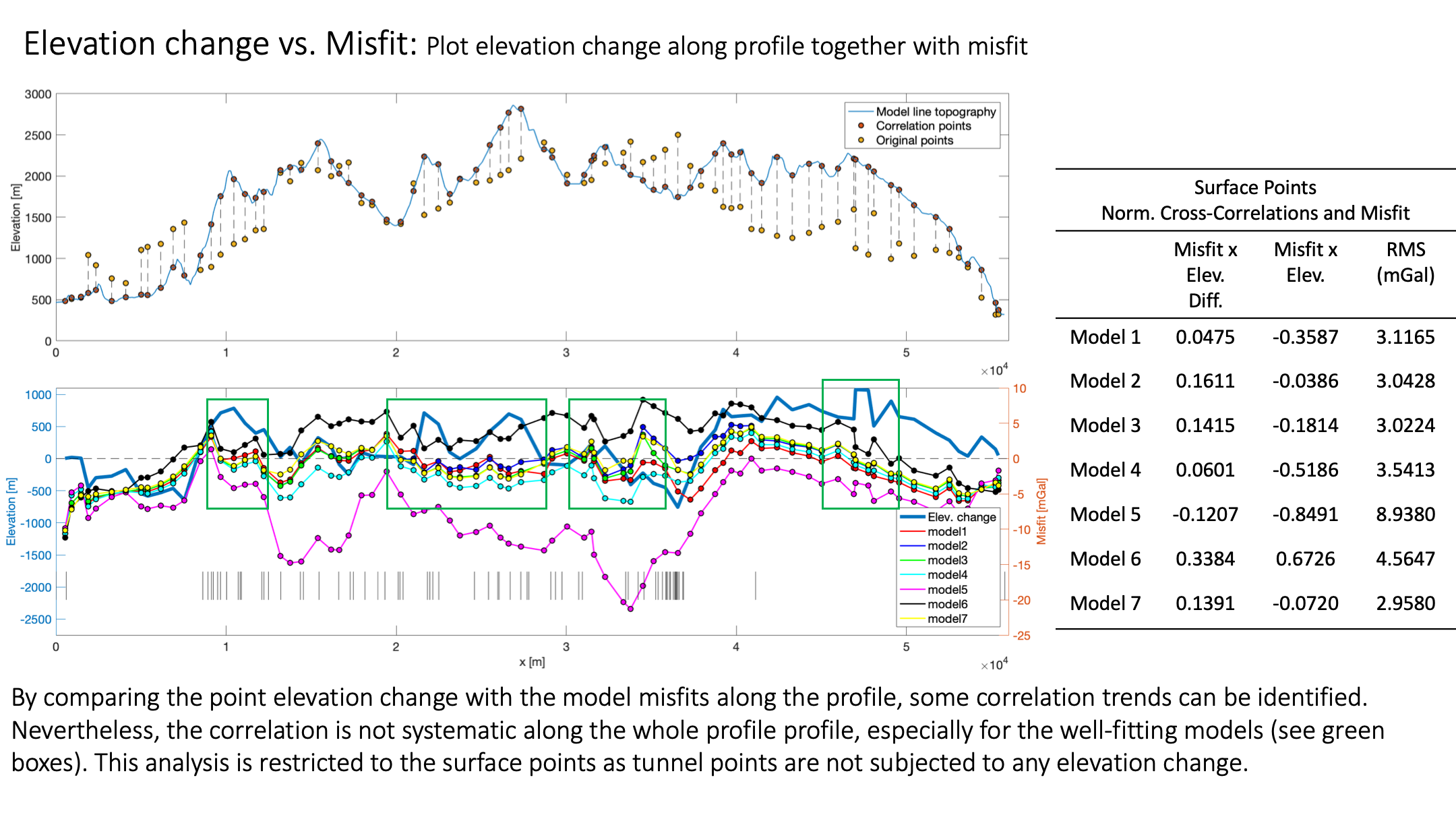

Supplement: Supplementary file 1 — Additional file 1. Top panel) 2D reference model topography; elevation of the original surface gravity measurement points (yellow) versus elevation of final 3D-to-2.5D-projected surface gravity measurement points (red). Bottom panel) Surface gravity model misfit versus 3D-to-2.5D-projection elevation change (blue thick line). Table) Zero-lag crosscorrelation between surface gravity model misfit and elevation difference, surface gravity model misfit and elevation; root-mean-square (RMS) misfit. [file 15_2022_422_MOESM1_ESM.png]

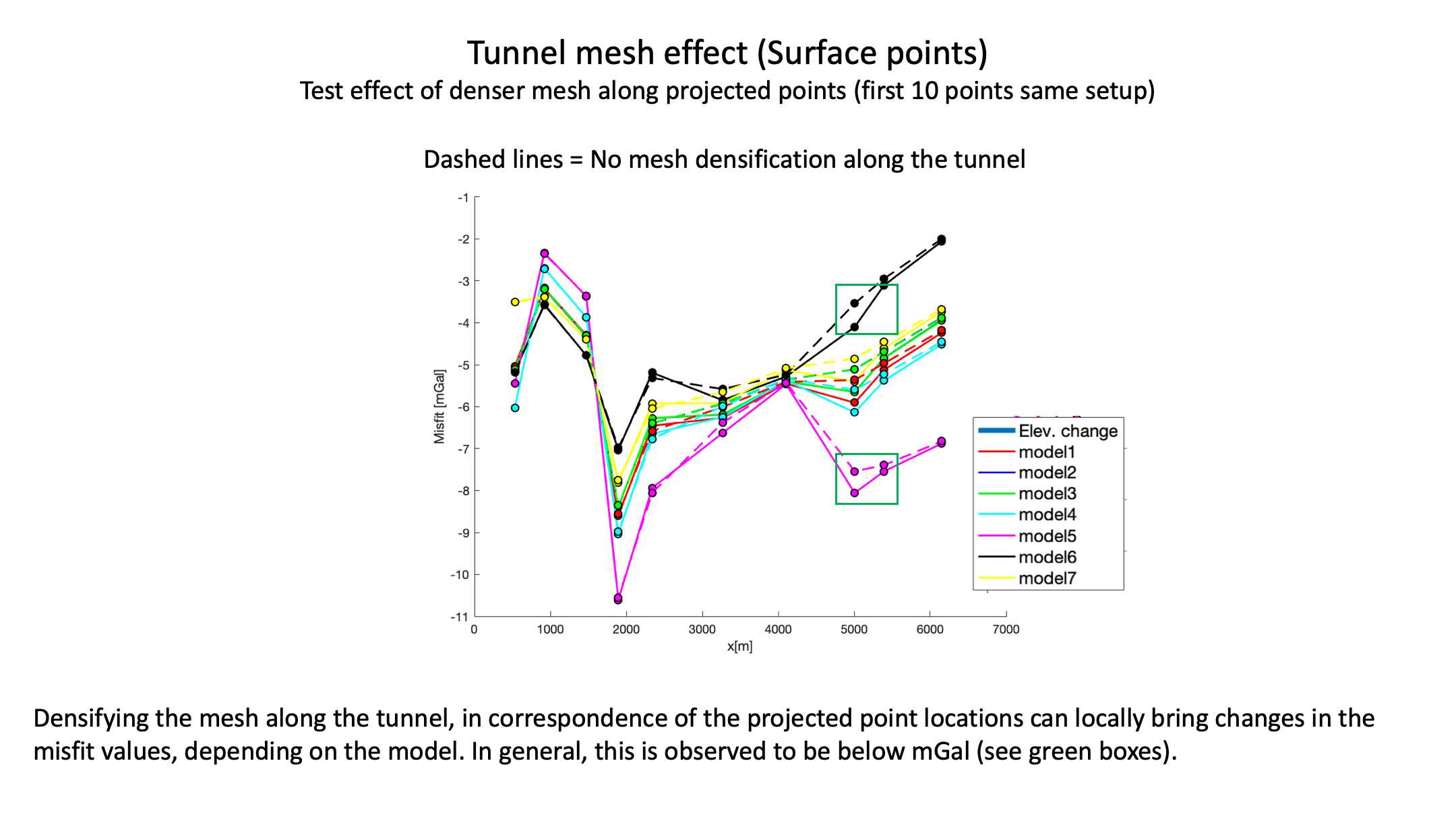

Supplement: Supplementary file 2 — Additional file 2. Surface gravity model misfit and associated variation (dashed lines) due to a 25% densification of the numerical mesh. These displayed points are located at the northern edge of the tunnel profile. [file 15_2022_422_MOESM2_ESM.png]
